# New Models for the Study of Ocular Toxoplasmosis. Comment on Ashander et al. Human Retinal Organoid Model of Ocular Toxoplasmosis. *Pathogens* 2025, *14*, 286

**DOI:** 10.3390/pathogens15040357

**Published:** 2026-03-27

**Authors:** Fabrizio Bruschi, Giovanni Casini

**Affiliations:** 1 Dipartimento Ricerca Traslazionale e Nuove Tecnologie in Medicina e Chirurgia, Università di Pisa, Via Savi 10, 56126 Pisa, Italy; 2 Department of Biology, University of Pisa, 56126 Pisa, Italy; giovanni.casini@unipi.it

*Toxoplasma gondii* is the causative agent of toxoplasmosis, a globally distributed zoonosis, which usually presents asymptomatically in immunocompetent individuals, unless the retina is affected [1].

Incidence and prevalence of ocular toxoplasmosis are difficult to quantify precisely as they depend on the overall prevalence of the infection in a given population and on the genotypes of *T. gondii* circulating in the area [1]. At present, *T. gondii* is considered the most common infectious agent of the retina worldwide, accounting for 20–60% of the overall cases presenting with posterior uveitis [2,3]. In South and Central America, for example, up to 50% of all cases of posterior uveitis can be attributed to toxoplasmosis [4]; this is particularly true in Brazil, where the reported seroprevalence for *T. gondii* is about 80%, ranging (depending on the region) from 6% to almost 18% in immunocompetent individuals, and is about 80% in children with congenital infection [5,6,7].

We read the paper by Ashander and colleagues (2025) with interest [8]. This paper is certainly innovative since it describes a new tool for the study of ocular toxoplasmosis in humans. However, it is to note that throughout the whole text, no limits of the described method are discussed; for example, although organoids have been described to be able to replicate, at least in part, the architecture and cellular complexity of the retina, the organoid sections shown in Figure 2 seem to lack a layered organization completely. Therefore, it is difficult to get any information about layer invasion by the parasite. In addition, similar studies using murine models are summarized only with a short sentence: “*Toxoplasmosis in humans varies in numerous ways from T. gondii infection in mice, the common experimental animal, with different routes of infection, pathogen recognition pathways, and cellular responses*”. The cited reference [9] refers to a study addressing the difference between mice and humans only considering the mechanism of innate immunity. Furthermore, Sher and colleagues state: “*rodents are more than just a convenient experimental model for studying infection with this protozoan pathogen.*” Finally, the sentence “*As an in vitro human-based model for studying ocular toxoplasmosis, retinal organoids would address several shortcomings of two-dimensional cultured cell and tissue explant models*” is not supported by any reference.

We were surprised that the authors completely disregarded the contribution of mouse studies and the different experimental models (see [10] for a review), including our paper describing a new ex vivo model based on mouse retinal explants [11]. Indeed, this method is quick to set up, requires only a relatively low number of animals, and offers easy manipulation and accessibility, allowing for different experimental conditions to be assessed, for example, the effects of putative drugs for therapy in the context of an intact retinal structure.

In conclusion, we recognize the importance of this new organoid-based model since it provides a setting closer to the human condition; however, the contribution of animal models should be acknowledged, and the advantages or drawbacks of the different approaches should be considered.

## References

[1] Fabiani S., Caroselli C., Menchini M., Gabbriellini G., Falcone M., Bruschi F. (2022). Ocular toxoplasmosis, an overview focusing on clinical aspects. Acta Trop..

[2] Holland G.N. (2003). Ocular toxoplasmosis: A global reassessment. Part I: Epidemiology and course of disease. Am. J. Ophthalmol..

[3] Montoya J.G., Liesenfeld O. (2004). Toxoplasmosis. Lancet.

[4] Vallochi A.L., Muccioli C., Martins M.C., Silveira C., Belfort R., Rizzo L.V. (2005). The genotype of *Toxoplasma gondii* strains causing ocular toxoplasmosis in humans in Brazil. Am. J. Ophthalmol..

[5] Aleixo A.L., Benchimol E.I., de Souza Neves E., Silva C.S.P., Coura L.C., Amendoeira M.R.R. (2009). Frequency of lesions suggestive of ocular toxoplasmosis among a rural population in the State of Rio de Janeiro. Rev. Soc. Bras. Med. Trop..

[6] Arantes T.E., Silveira C., Holland G.N., Muccioli C., Yu F., Jones J.L., Goldhardt R., Lewis K.G., Belfort R. (2015). Ocular Involvement following postnatally acquired toxoplasma gondii infection in Southern Brazil: A 28-year experience. Am. J. Ophthalmol..

[7] De Angelis R.E., Veronese Rodrigues M.D.L., Passos A.D.C., Bollela V.R., Freitas e Silva M.S., Vieira B.R., de Lucena M.M., Moralles T.D., de Morais Vicente L., de Melo Rocha G. (2021). Frequency and visual outcomes of ocular toxoplasmosis in an adult Brazilian population. Sci. Rep..

[8] Ashander L.M., Lidgerwood G.E., Lumsden A.L., Furtado J.M., Pébay A., Smith J.R. (2025). Human Retinal Organoid Model of Ocular Toxoplasmosis. Pathogens.

[9] Sher A., Tosh K., Jankovic D. (2017). Innate recognition of Toxoplasma gondii in humans involves a mechanism distinct from that utilized by rodents. Cell. Mol. Immunol.

[10] Rodriguez-Fernandez V., Casini G., Bruschi F. (2021). Ocular Toxoplasmosis: Mechanisms of Retinal Infection and Experimental Models. Parasitologia.

[11] Rodriguez-Fernandez V., Amato R., Piaggi S., Pinto B., Casini G., Bruschi F. (2024). A New Ex Vivo Model Based on Mouse Retinal Explants for the Study of Ocular Toxoplasmosis. Pathogens.

